# Pharmacotherapy and Nutritional Supplements for Neovascular Eye Diseases

**DOI:** 10.3390/medicina59071334

**Published:** 2023-07-20

**Authors:** Dario Rusciano, Paola Bagnoli

**Affiliations:** 1 Fidia Ophthalmics, 95125 Catania, Italy; 2 Department of Biology, University of Pisa, 56123 Pisa, Italy; paola.bagnoli@unipi.it

**Keywords:** eye, retina, neo-angiogenesis, drugs, nutraceuticals

## Abstract

In this review, we aim to provide an overview of the recent findings about the treatment of neovascular retinal diseases. The use of conventional drugs and nutraceuticals endowed with antioxidant and anti-inflammatory properties that may support conventional therapies will be considered, with the final aim of achieving risk reduction (prevention) and outcome improvement (cooperation between treatments) of such sight-threatening proliferative retinopathies. For this purpose, we consider a medicinal product one that contains well-defined compound(s) with proven pharmacological and therapeutic effects, usually given for the treatment of full-blown diseases. Rarely are prescription drugs given for preventive purposes. A dietary supplement refers to a compound (often an extract or a mixture) used in the prevention or co-adjuvant treatment of a given pathology. However, it must be kept in mind that drug–supplement interactions may exist and might affect the efficacy of certain drug treatments. Moreover, the distinction between medicinal products and dietary supplements is not always straightforward. For instance, melatonin is formulated as a medicinal product for the treatment of sleep and behavioral problems; at low doses (usually below 1 mg), it is considered a nutraceutical, while at higher doses, it is sold as a psychotropic drug. Despite their lower status with respect to drugs, increasing evidence supports the notion of the beneficial effects of dietary supplements on proliferative retinopathies, a major cause of vision loss in the elderly. Therefore, we believe that, on a patient-by-patient basis, the administration of nutraceuticals, either alone or in association, could benefit many patients, delaying the progression of their disease and likely improving the efficacy of pharmaceutical drugs.

## 1. Introduction

In order to properly frame the content of this review, which deals with the treatment of neovascular eye diseases by pharmaceuticals and/or nutraceuticals, we begin by highlighting two main considerations. 

The first point to consider is that the molecular mechanisms underlying any kind of pathology, including those discussed in this review, are extremely complex and cannot be described as a simple cascade of events. Instead, they are better defined by a network of interactions containing several critical nodes, each of which might represent a potential therapeutic target. On a larger scale, human diseases could also be represented by a network with thousands of nodes interconnecting all the pathologies with one another at different levels [1]. However, for the sake of simplicity, we have sometimes neglected this level of complexity, dealing with the different causative elements (each one a ‘node’ in the pathology network) of neovascular retinal diseases in partial isolation.

The second point to consider is that we have chosen to use the term ‘nutraceutical’ in accordance with the description originally given by De Felice in 1995 [2], in which he described a nutraceutical as “a food or part of a food that provides medical or health benefits, including the prevention and/or treatment of a disease.” In fact, while food supplements are used as “an aid for the body but are not required to have a proven clinical efficacy on a health condition,” nutraceuticals are expected to influence the health status of the subjects using them [3]. 

Visual impairment among the elderly is a major health problem. With advancing age, the normal function of tissues in the eye may degenerate, thus increasing the occurrence of ocular pathologies. Accordingly, demographic studies have shown that age is the best predictor of blindness and visual dysfunction [4]. The most common causes of age-related visual impairment in the elderly are damages to the anterior segment of the eye, whereas in its posterior segment neovascular diseases account for a high percentage of visual dysfunctions [5].

Critical vision loss generally originates from the late diagnosis of eye diseases because of the limited compromise of visual performance at the early stages of the pathology. In this respect, early detection of functional or morphological alterations in patients at risk may allow for the prevention of permanent visual loss and intervention with less aggressive treatments to halt or at least delay disease progression. In this respect, encouraging evidence of the benefits of diet supplementation derives from a steadily increasing number of published preclinical studies, although the paucity of prospective, randomized human clinical trials makes the impact of diet supplements still poorly accepted by ophthalmologists, thus resulting in a low prescription rate [6,7]. On the other hand, increasing evidence from preclinical models also demonstrates the efficacy of novel pharmacological therapies to treat neovascular retinal diseases, although in this case, more clinical trials are necessary in order to move the novel formulations from bench to bedside [8,9]. 

Therefore, in order to propose and support a new point of view, in the present review we intend to address and highlight the possible cooperation—to be implemented in medical practice—among the four factors that must be considered in the management of a pathology: risk prediction, preventive treatments, early diagnosis, and innovative treatments. Among them, preventive medicine expenditure in Europe accounted for on average about 3% of the total health expenditure in 2020 [10]. Europe in particular is paying a heavy price for chronic diseases, of which diabetes complications, including proliferative retinopathies, affect over one-third of all people with diabetes and constitute 80% of causes of vision loss [11]. In fact, too often, patients at risk are only kept under observation until they develop the early signs of the pathology, and no pharmacologic therapy is usually given before a functional disease is manifest. This logically happens because the speed of pathology progression is usually slow and unpredictable, drug therapies are costly, and they may have side effects, so patients are not given treatments of any type in cases of asymptomatic diseases. 

Alternatively, we propose that already in the very early stages, even when only risk factors are present, preventive treatments with adequate nutraceuticals might be established in order to delay, as much as possible, the development of the early manifest signs of the disease. At this time, new, less aggressive therapies might be added to the supplement regimen. Indeed, even though by definition diet supplements cannot be therapeutic by themselves, in the case of a slow-progressing disease, they could be seen as a useful and even necessary complement to pharmacologic therapy, in that they usually have a different function and could even decrease the side effects of such therapies. However, exploiting nutraceuticals in the prevention/early treatment of a disease implies the enhancement of prevention medicine over curative medicine, a goal still far from being reached. 

There is increasing evidence that age-related diseases are correlated with oxidative stress-derived inflammatory conditions, characterized by an imbalance between the generation of reactive oxygen species (ROS) and the antioxidant potential. In this respect, oxidative stress and inflammation can be considered the early causative players in the increasing incidence of chronic diseases (including eye pathologies) associated with advanced age.

The eyes—and especially the retina—are usually exposed to a series of constant and progressive oxidative events because of their high metabolic activity, which also includes the biochemical cascade of phototransduction. Therefore, due to the prevalence of oxidative stress over antioxidant protection, the retina may progressively degenerate, developing different pathologies depending on peculiar environmental conditions and innate individual susceptibility. Oxidative stress activates a variety of transcription factors, which lead to the expression of genes involved in inflammatory processes that reverberate on oxidative stress itself, thus triggering a feedback loop.

The pathologies macular degeneration, diabetic retinopathy, and retinopathy of prematurity all recognize among their causes an excess of oxidative stress not compensated by antioxidant defense. Therefore, a logical approach to the prevention and treatment of such pathologies would be to increase the antioxidant defense of the retina via local or systemic administration of adequate antioxidants. Such treatment should also be maintained in case the pathology progresses to a sight-threatening disease, as a subsidiary to the specific drug therapies eventually prescribed by the ophthalmologist.

In this review, we will limit our focus to the treatment of proliferative retinal diseases, which are the most difficult to treat and for which no definite therapy exists yet. To this purpose, we explored the internet by using combinations of relevant keywords, such as neovascular retinopathies, therapeutic treatments, food supplements and/or nutraceuticals, and disease progression. A selection of the most relevant publications appearing in high-impact international journals has been chosen and used to build this comprehensive review. 

## 2. Proliferative Retinopathies

The growth and invasion of new blood vessels into the retina is the consequence of prolonged oxidative stress and inflammation, which may weaken the protective barriers and cause local ischemic events, thus triggering the production of angiogenic factors. Therefore, the use of treatments aimed at contrasting oxidative stress, inflammation, and pro-angiogenic factors is an available therapeutic option to prevent the chain of events leading to neo-angiogenesis [12,13]. 

Neovascular retinal pathologies include proliferative diabetic retinopathy (PDR) and retinopathy of prematurity (ROP), which are characterized by leakage and/or neo-angiogenesis derived from retinal vessels; and subretinal vascular diseases, including the wet or neovascular form of age-related macular degeneration (nAMD), which is characterized by new vessels invading from the choroid into the avascular outer retina and subretinal space. 

Persistent values of high blood sugar are the hallmark of badly controlled diabetes. The enduring high blood sugar activates several interconnected oxidative and inflammatory pathways that play a prominent role in triggering angiogenic processes leading to increased vascular permeability through blood retinal barrier (BRB) damage, which is the basic condition preluding to PDR [14]. Excessive accumulation of ROS—not compensated by the antioxidant defense system—induces mitochondrial damage, cellular apoptosis, inflammation, and lipid peroxidation, finally resulting in structural and functional alterations in the retina [15]. In particular, injury to pericytes and thickening of the capillary basement membrane result in dysregulation of the blood flow within the retina and the development of microaneurysms. As capillaries are occluded, retinal ischemia occurs, resulting in the release of angiogenic factors such as vascular endothelial growth factor (VEGF), which stimulates endothelial cell proliferation and disrupts endothelial tight junctions, thus increasing vasopermeability. The subsequent neovascularization on the surface of the retina can finally lead to vitreous hemorrhage and tractional retinal detachment.

DR is a growing problem in both types of diabetes (I and II) because of the aging-associated decrease in blood flow, retinal thinning, and microglial changes that render the retina more vulnerable to oxidative and ischemic damage [16,17]. Years after diabetes onset, the severity of DR progresses while adaptive changes begin to fail, leading to PDR, characterized by retinal vascular endothelial cell proliferation and invasion of retinal vessels through the internal limiting membrane into the vitreous. A recent study on more than 70,000 patients with type 2 diabetes found that 5 years after the initial diagnosis, 1.74% of patients had developed PDR [18]. Proliferating vessels may damage the vitreous and the retina, as said before. In concomitance, BRB leakage may lead to diabetic macular edema (DME), a major cause of dramatic visual dysfunction that affects about 5.5% of diabetic patients, as assessed by a recent meta-analysis of clinical reports [19].

An additional retinal pathology in the elderly is age-related macular degeneration (AMD). AMD may occur in different forms: an atrophic or ‘dry’ form, a neovascular or ‘wet’ form (nAMD), or a mixture of both, all resulting in partial or complete loss of central vision [20]. Atrophic AMD is the initial stage of the disease and is characterized by the thickening of Bruch’s membrane (BrM), the membrane that separates the retinal pigment epithelium (RPE) from the choroid, and through which all metabolic exchanges occur. Catabolic waste products (lipids and proteins) accumulate within the space between the RPE and the BrM, leading to the formation of deposits called drusen [21], which impair the metabolic exchanges necessary to the survival of RPE cells and photoreceptors. Drusen not only make a mechanical barrier but also activate the natural immune system [22], inducing local inflammation. In genetically predisposed individuals, drusen may start an inflammatory process, involving natural immunity, and specifically the complement cascade, which can, in the end, lead to the rupture of the BrM, so that capillary vessels from the choroid invade the retina, causing blood and fluid to leak into the macula [23]. Genetic tests have thus been developed that take into account the different risk factors for AMD, including age, body mass index, smoking habit, familiarity, and some single nucleotide polymorphisms mainly involving complement elements, and correlate with the probability of developing AMD [24]. However, knowing the individual risk for the development or progression of AMD would make little sense if nothing could be done in response to this knowledge. Hence, it becomes critical for physicians and their patients to know whether some preventive treatments exist that are able to decrease the risk of the development/progression of AMD, such as, for instance, the use of some nutraceuticals [25]. In fact, the dry form in its early stages often goes unnoticed, because the patient suffers a very limited compromise of the central vision and does not seek medical advice. As no curative treatments are currently available for dry AMD, many studies are being conducted to find effective drugs to prevent the appearance of drusen, thus counteracting the progression towards the wet form [23].

As a counterpart of aging-associated neovascular retinal diseases, retinopathy of prematurity (ROP) is characterized by an abnormal growth of retinal blood vessels in preterm newborns. An increasing incidence of premature births and the high standard of neonatal care are leading to an ever-increasing survival rate of low and very low birthweight infants that are highly susceptible to ROP. Recent epidemiological surveys about global ROP cases reported that roughly 190,000 preterm infants were suffering from any stage of ROP, among whom 20,000 became totally blind or developed severe vision loss. However, ROP is considered a preventable and treatable retinal disease [26]. At birth, sudden oxygen exposure of the hypoxic newborn followed by supplemental oxygen administration halts the development of the immature retinal vasculature, leading to retinal ischemia, thereby resulting in increased levels of angiogenic and inflammatory factors. Angiogenesis and inflammation in turn lead to hypoxia-induced neovascularization that, in some cases, can cause permanent vision problems or even blindness during childhood [27]. 

## 3. Animal Models

A large body of our knowledge on the molecular pathogenesis of neovascular retinal diseases stems from animal models [28]. It is, however, recognized that they cannot fully reflect the complex human conditions, thus emphasizing the necessity and importance of clinical studies with natural, spontaneous physiological behavior. Among animal models of retinal eye diseases, the rodent model of streptozotocin (STZ)-induced DR very closely recapitulates the pathologic events occurring in the early phase of human DR, though not including the late proliferative stage, probably owing to the short lifespan of the animal model and thus the shorter duration of the disease [29]. In this respect, the oxygen-induced retinopathy (OIR) model, which is generally used to mimic retinopathy of prematurity (ROP) in preterm infants, is also adopted to imitate the proliferative phase of DR. The OIR model is based on the exposure of mouse pups to hyperoxia during a phase when their retinal vasculature is still developing [30]. This leads to capillary depletion, and upon return to room air and normal oxygen tension, it results in retinal ischemia and abnormal proliferation of the retinal vasculature in response to hypoxia that upregulates HIF-1 and its major transactivated gene, VEGF. Numerous studies using the OIR model have revealed that the dysregulation of angiogenic factors and the influence of inflammatory processes play a pivotal role in the vascular pathogenesis that characterizes the proliferative phase of DR. 

The neovascular phase of AMD is characterized by the abnormal growth of vessels from the choroidal vasculature into the neurosensory retina through ruptures of the BrM. Such choroidal neovascularization (CNV) represents the late phase of exudative AMD (nAMD), an eye disease that blurs central vision. It happens when aging-related inflammatory events, a consequence of oxidative stress, cause damage to the macula. Although the prevalence and need to develop effective treatments for AMD have led to the development of multiple animal models, none of them yet recapitulates all of the features of human AMD. Animal models based on artificial laser damage of the BrM and obliteration of retinal vessels, thus inducing local ischemia and the production of inflammatory and angiogenic factors, mimic choroidal neovascularization, and have served as the backbone to test new therapies [31]. 

## 4. Management of Neovascular Retinal Diseases

**Figure 1:**
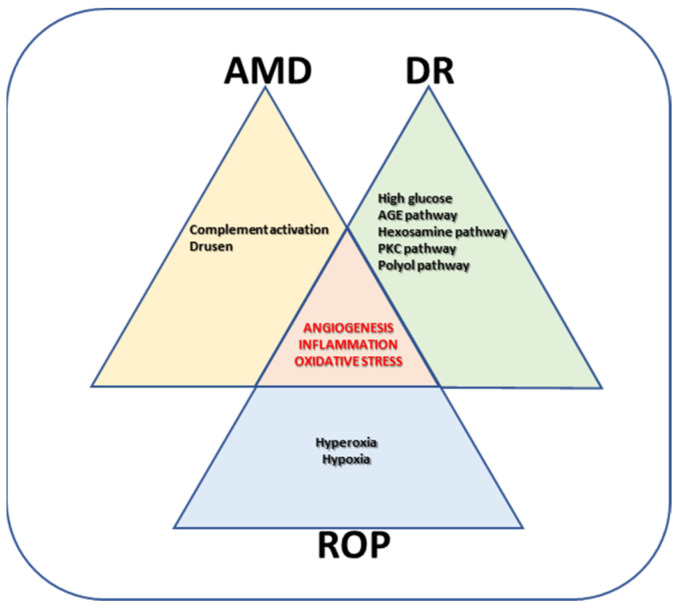
A schematic drawing illustrating the common elements (center, red) of the three main retinal neovascular diseases dealt with in this review. Oxidative stress, inflammation, and angiogenesis also represent the main targets of pharmacological therapies or nutraceutical treatments. AMD (Age-Related Macular Degeneration); DR (Diabetic Retinopathy); ROP (Retinopathy of Premature).

As shown in the schematic representation in Figure 1, proliferative retinopathies share some etiologic factors, among which inflammatory cytokines and angiogenic factors are the main triggers of retinal damage, finally leading to permanent vision impairment or loss. Therefore, they also represent the main targets for therapeutic treatments. These elements can be targeted by specific drugs, usually given by local intravitreal administration, although laser photocoagulation and vitreoretinal surgery are also used in the case of drug unresponsiveness.

### 4.1. Laser Photocoagulation and Vitrectomy

Until recently, the management of ocular neovascular pathologies mainly relied on surgical or para-surgical invasive treatments, which can control pathological neovascularization, but may also impair vision and hardly guarantee to stop the progression of the pathology [32]. Laser photocoagulation and vitrectomy have been the standard of care for PDR for decades. In particular, the thermal destruction of the ischemic retina decreases the signal for angiogenesis, promotes regression of neovascularization, and decreases the risk of hemorrhage or the development of tractional membranes. However, panretinal photocoagulation (PRP) has well-established limitations, as peripheral vision is permanently lost with the destruction of the peripheral retina. Among side effects, the probability of developing macular edema after PRP has a major impact since laser-associated breaks in Bruch’s membrane can lead to the development of choroidal neovascularization.

Cryotherapy and photocoagulation are current effective ablation therapies of the pathological neovessels in ROP, but systemic and functional side effects have been reported [33], such as a higher risk of cataract, myopia, cornea, and lens burns [34]. Recently, intravitreal injection of anti-VEGF agents has become a new approach for treating ROP, yet it is not devoid of risks and side effects [35]. The recent discovery that the β-adrenergic system contributes to hypoxia-induced retinal neovascularization and that treatment with the unselective β1/β2 adrenoceptor antagonist propranolol ameliorates retinal vessel proliferation kicked off a series of clinical trials using propranolol in preterm infants [36], although the ethical difficulty of enrolling premature infants and the practical issues in the design and conduct of the trial have delayed the transfer of propranolol from bench to bedside.

### 4.2. Photodynamic Therapy

Thermal photocoagulation is limited in its use to cases of choroidal neovascularization (CNV) that do not involve the fovea. Therefore, it is applicable to only a reduced subset of patients, with the caveat that it can result in an immediate loss of visual acuity. Photodynamic therapy (PDT) was originally developed to treat vascularized tumors and is based on the use of the photosensitizer verteporfin, which is photochemically activated by laser light, thus destroying the blunt neovessels in which it accumulates [37]. Preclinical and clinical evidence has indicated that this photodynamic approach can be used to treat the neovascular form of age-related macular degeneration with good efficacy and safety. The normal choroidal neovasculature is only marginally affected by this treatment, causing only minimal damage to the neurosensory retina and thus not inducing a permanent loss of visual acuity. Finally, it must be said that this therapy, although considered safe enough, is not devoid of possible side effects. In a limited retrospective study on 52 patients (58 eyes, 140 treatments), it was reported that acute exudative maculopathy (AEM) appeared to be a rare (incidence 1.4% per treatment) and unpredictable reaction related to the proinflammatory effects of PDT. Because of the inherent limitations of the study, the true incidence of AEM after PDT might be even higher [38].

### 4.3. Pharmacological Therapies

Although strategies to inhibit VEGF have proven to be highly successful in some clinical studies, there remains the possibility of significant adverse effects regarding the block of crucial physiological roles of VEGF and the invasive nature of the treatments given by intravitreal injection. In addition, other pro-angiogenic factors, which play important roles in the development of these diseases, are not eliminated and may contribute to the failure often observed with anti-VEGF therapies [39]. Moreover, the ascertained role of inflammatory pathways in the etiology of neovascular ocular diseases prompted the use of intraocular corticosteroids given by repeated intravitreal injections, such as in the case of triamcinolone acetonide [40] or by intravitreal implants of dexamethasone or fluocinolone [41]. However, a conclusive therapy is far from being reached, and new types of effective treatments are required, including their route of delivery, which directly impacts the degree of immune and inflammatory reactions. In this respect, subretinal delivery produces a weaker humoral response than the intravitreal route, but induces a stronger inflammatory reaction [42]. Finally, the potential efficacy of novel therapies, including gene therapy, is under scrutiny, as the eye is one of the few organs of the body for which gene therapy has received FDA approval [43]. In this respect, targeting HIF-1, a master regulator of angiogenesis, through siRNA-mediated HIF-1α gene silencing is expected to provide a novel and attractive therapeutic strategy to fight neovascular ocular diseases [44]. 

### 4.4. Intraocular Anti-VEGF Therapy

Finding safer and more efficient anti-angiogenic/anti-inflammatory drugs is still a key challenge for the treatment of proliferative retinopathies [45]. To this end, new therapeutic approaches need to be developed that could be used either alone or in association with other currently available treatments. In line with this need, a growing body of evidence supports novel therapies based on the involvement of an increasing number of angiogenic-related factors as possible targets, but their development is slowed down by a conservative pharmaceutical market, which moves with great difficulty from the current therapies. 

In recent years, as the pathogenic role of VEGF in DME has been well recognized, the intravitreal injection of anti-VEGF antibodies has become the first line of treatment for DME [46] and nAMD [47]. Currently, anti-VEGF therapies are mainly based on intravitreally administered anti-VEGF monoclonal antibodies. To mention the most popular on the market, bevacizumab is a monoclonal antibody, and ranibizumab is a monoclonal antibody fragment, both binding and neutralizing VEGF-A. Remarkable progress has then been made towards the design of novel ocular therapeutics with enhanced activity and minimal toxicity to the ocular tissue. Among the most widely used drugs, the recently approved aflibercept belongs to the group of recombinant decoy receptors for VEGF. It sequesters free VEGF, thus preventing signal transduction through its cognate receptors, VEGFR1 and/or VEGFR2, thereby blocking the pro-inflammatory, edemigenous, and pro-angiogenic effects of VEGF. Aflibercept seems to be as effective as the monoclonal antibodies bevacizumab and ranibizumab, but it may be administered less frequently. As recently reviewed [48], aflibercept is commonly used in the therapy of DME patients, resulting in greater visual acuity improvement and anatomic restoration than traditional laser therapy or monoclonal antibody drugs. The recent CLARITY clinical trial demonstrated that in patients with PDR, the topographical response of retinal neovascularization to aflibercept treatment was superior to panretinal photocoagulation [49]. Regarding the pharmacokinetics (PK) of anti-VEGF treatments, the intravitreous half-life of aflibercept in humans, as predicted by a mathematical model, is approximately 7 days, which is longer than that of ranibizumab and similar to that of bevacizumab [50]. This kinetics reasonably accounts for a drug efficacy that does not differ between aflibercept and bevacizumab, at least for patients with myopic CNV [51]. However, in 1 year of DME treatment, the incremental cost-effectiveness ratio of aflibercept is higher than that of bevacizumab, though it is still lower than that of ranibizumab [52]. In conclusion, since the advent of the therapeutic use of anti-VEGF compounds in the early 2000s, patients with DR have had new hope of vision-improving therapies that do not rely on destructive laser treatments, and nAMD went from a nearly untreatable sentence of blindness to a much more manageable condition with early intervention. Nonetheless, a certain number of patients remain unresponsive to anti-VEGF treatments, with a dramatic progression of the disease [53]. Indeed, a more recent study has determined that more than 30% of patients tend to lose more than 15 letters in the 10 years after treatments [54].

More recently, a new class of engineered antibodies has been produced. Among others, brolucizumab is a humanized, single-chain variable fragment binding the three major isoforms of VEGF-A (VEGF-110, VEGF-121, and VEGF-165). It has been given by intravitreal injection to patients with neovascular age-related macular degeneration (nAMD) who previously showed an incomplete response to the classical anti-VEGF treatments, with the result of significantly reducing their choroidal thickness [55]. Faricimab is a bispecific monoclonal antibody that inhibits both VEGF-A and angiopoietin 2 (Ang-2), responsible for two different pathways in retinal angiogenesis, and preliminary clinical data obtained in the treatment of nAMD suggest that its use might extend treatment intervals to up to 16 weeks, with respect to the 4–8–12 weeks required by the other antibodies [56].

RNA-based therapies are currently being explored as alternative therapeutics for the management of proliferative retinopathies due to their role in the regulation of VEGF gene expression [57]. In particular, RNA interference therapy inhibits the activity of the VEGF gene at the post-transcriptional level and reduces its protein expression with high specificity. In preclinical studies using the OIR model, bioreducible lipidoid nanoparticles conveying VEGF siRNA have been shown to effectively inhibit retinal vessel proliferation after intravitreal injection by reducing the expression of VEGF mRNA and protein [58]. Bevasiranib is a prototype of such molecules and is a siRNA-based anti-angiogenic agent proposed for the intravitreal treatment of neovascular AMD by specific targeting of VEGF gene expression [59]. 

Despite the convincing therapeutic effects of anti-VEGF agents, this treatment is being criticized in view of the function of VEGF in maintaining neurons as well as its action as a trophic factor. In fact, recent experimental data indicate an autocrine role of VEGF in sustaining growth and survival of RGC, which is consistent with clinical observations showing that glaucomatous patients injected with anti-VEGF because of AMD or DME comorbidity displayed a significant reduction of RGC axon fiber layer thickness [60]. More clinical data support the relationship between repeated anti-VEGF injections and the development of ocular hypertension or glaucoma [61,62]. Moreover, very recent preclinical data suggest that the effects of VEGF in DR can be modulated by the concomitant presence of NGF [63]. Therefore, regulating factors upstream of VEGF have received attention as a desirable therapeutic approach to eliminating off-target effects. 

During the last decade, the three popular anti-VEGF drugs, bevacizumab, ranibizumab, and aflibercept, have been subjected to clinical trials to establish their efficiency, bioavailability, and functional results in children affected by ROP [64,65,66,67]. In fact, increased VEGF in response to the ischemic condition following premature oxygen exposure in preterm infants causes pathological vessel proliferation that can be treated with intraocular anti-VEGF therapy that is, however, not devoid of risks and side effects. On the one hand, ocular complications such as cataracts and retinal scarring could be prevented, but on the other, reduced systemic VEGF expression may result in a higher risk of delayed neurodevelopment and abnormal neurobehavior in treated babies [35].

### 4.5. Steroid Intravitreal Implants

In DME, Muller cells are the first to be affected, and with the progression of the disease, they may become gliotic, thus triggering drastic inflammatory events. Activated Müller cells release cytotoxic mediators that are responsible for the recruitment of leukocytes, blood–retinal barrier breakdown, direct glial dysfunction, and neuronal cell death. Corticosteroids work by suppressing the multiple inflammatory genes that are activated in chronic inflammatory diseases, such as DME, mainly by reversing histone acetylation of their promoters via the binding of activated glucocorticoid receptors to coactivators and the recruitment of the enzyme histone deacetylase-2 to the activated transcription complex [68]. 

Targeting inflammatory processes and preventing Muller cell gliosis is the rationale for DME treatment with DEX implants at an earlier stage of the disease. Steroids released by an implant last longer, reducing the burden of frequent clinical visits while on VEGF inhibitor treatments [69]. They may also treat the inflammatory component of DME more effectively, thus targeting a considerable proportion of patients not responding satisfactorily to anti-VEGF agents, even with intensive treatment over the first year. Steroid intravitreal implants have demonstrated efficacy in improving visual acuity and reducing retinal thickness even in DME eyes that are refractory to anti-VEGF treatments [70]. In patients responding to anti-VEGF therapy, steroid implants allowed a relevant decrease in anti-VEGF intravitreal injections per year (up to 11 injections) without major side effects, except for an increased risk of IOP elevation in about 3% of DME patients [71].

### 4.6. Systemic Therapies: Preclinical Evidence of Novel Treatments

Among the therapeutic approaches, systemic therapies might reduce the adverse effects of repeated intravitreal administrations, but would be limited by major problems, including their poor diffusibility to the eye as well as the safety/side effects of drug administration through the systemic route. Therefore, no systemic therapies are on the market yet, with the exception of those for intensive control of hyperglycemia and hypertension. 

An increasing number of therapeutic approaches that are systemically administered are being tested at the preclinical level, and several research groups are actively pursuing research projects to determine the efficacy and safety of multi-factorial drugs targeting eye neovascular pathologies. To name only a few among the many putative drugs that have been tested preclinically in animal models of DR, systemic administration of bradykinin (BK) receptor antagonists has been shown to improve altered retinal vascular permeability by counteracting diabetes-associated oxidative stress [72,73]. In addition, in a mouse model of OIR, the i.p. injection in mouse pups of the BK antagonist fasitibant has been shown to block the signaling from the BK receptor 2 (BK2R), thus delaying retinal vascularization and indicating that the retinal endothelium is a target of the BK/BK2R system [74]. Other preclinical studies in models of neovascular eye diseases have shown that the systemic administration of AKB-9778, a small-molecule agonist of the angiopoietin receptor Tie2, stabilizes the vasculature against multiple permeability factors, thus reducing vascular leakage and retinal neovascularization. This effect has been confirmed by clinical trials in DME patients in which the subcutaneous injections of AKB-9778 gave additional benefits to VEGF suppression [75,76,77]. The efficacy in preclinical trials of the systemic administration of lipoprotein-associated phospholipase A2 inhibitors in ameliorating BRB damage during DR [78] has emphasized the treatment with statins as a possible treatment for DR because of their association with dyslipidemia. Although epidemiologic studies have failed to reveal the consistency of statin therapy, two recent randomized clinical trials have demonstrated the beneficial effects of fenofibrate therapy (lowering blood lipids by a mechanism different from statins) in reducing the progression of DR independently of its effect on serum lipid levels [79]. Among systemic drugs, preclinical evidence has demonstrated an important role for the inhibitors of the urokinase-type plasminogen activator receptor (uPAR) system in animal models of neovascular disease of the eye. The binding of uPA to uPAR is instrumental for the activation of plasminogen to plasmin, which, in turn, initiates a series of proteolytic cascades to degrade the components of the extracellular matrix, finally leading to endothelial cell proliferation. Investigations on the efficacy of uPAR inhibitors started with the demonstration that the uPAR system acts in the retina as a key player at the intersection between angiogenesis, inflammation, and neurodegeneration. In particular, uPA and its receptor uPAR, by interacting with the formyl peptide receptor (FPR), play a central role in the proteasome system that participates in matrix protein degradation, thus allowing endothelial cell migration [80]. Along this line, the identification of a novel drug inhibiting uPAR interaction with FPR has been the cue for a series of preclinical investigations that concurred to demonstrate that systemic administration of a distinct uPAR inhibitor, UPARANT or Cenupatide, counteracts the major pathological signs of experimental DR, including angiogenesis, neovessel permeability, inflammation, and retinal cell degeneration [81]. Successful demonstration of the efficacy of the UPAR system blockade allowed us to suggest UPARANT as a potential and potent alternative approach to delay DR progression and/or as an adjunctive therapy in combination with intravitreal anti-VEGF drugs with the aim of increasing their efficacy. Most recently, the multi-kinase inhibitor sorafenib, originally developed to fight kidney and liver cancers, has been formulated in nanotechnological eye drops. In such a formulation and administered topically on the eye, it has been shown to be able to treat preclinical models of diabetic retinopathy, choroidal neovascularization, and retinal ischemic damage due to elevated IOP [82].

Pharmacological approaches have been recently directed toward HIF-1 because of its major role in angiogenic pathways. HIF-1 is a transcription factor consisting of an oxygen-regulated α-subunit and a constitutively expressed β-subunit. Under normal oxygen concentrations, HIF-1α is hydroxylated by prolyl hydroxylases (PHDs) using molecular oxygen and 2-oxoglutarate as co-substrates. Subsequently, hydroxylated HIF-1α is recognized by the tumor-suppressing protein von Hippel–Lindau and then polyubiquitinated for degradation by proteasomes. Under hypoxic conditions, however, the function of PHD is limited, and the stabilized HIF-1α dimerizes with HIF-1β for translocation into the nucleus to activate the transcription of target genes, including VEGF. The role of the HIF-VEGF axis in the eye has been studied over the last 10 years, and it has become clear that HIF actively participates in the pathogenesis of many ocular diseases [83]. Therefore, the development of novel HIF-1α inhibitors targeting pathological angiogenesis in ocular diseases is strongly pursued at the preclinical level. In experimental models, for instance, anti-HIF-1α siRNA prior to hypoxia exposure has been shown to decrease HIF-1α and VEGF expression and reduce hypoxia-induced angiogenesis, suggesting that the activated HIF-1α/VEGF pathway is responsible for hypoxia-induced angiogenesis [84]. In addition, selected compounds inhibiting HIF-1α have been investigated on screening platforms. Their anti-HIF activity after oral administration resulted in a significant suppression of retinal neovascular tufts in the OIR model [85]. Administration of HIF-1-specific inhibitors, previously demonstrated to counteract neovascularization in experimental models, are currently in clinical trials, indicating their feasibility as therapeutic strategies [86]. 

**Figure 2:**
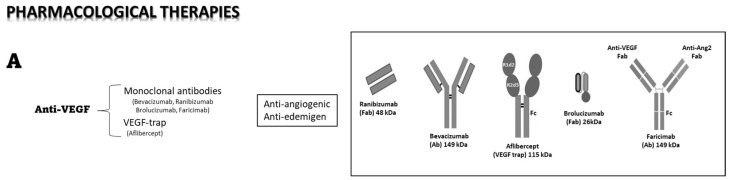

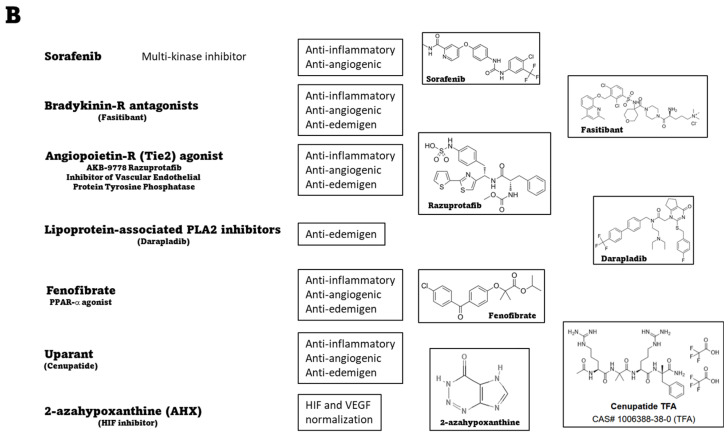
A schematic drawing illustrating some pharmacological therapies for neovascular retinal diseases. (**A**) Anti-VEGF strategies. (**B**) Alternative strategies.

Figure 2 below reports some of the recent therapeutic developments.

## 5. Nutraceuticals: Which Place in the Management of Neovascular Eye Diseases?

In addition to drug medications, dietary supplements may also contribute to the control of proliferative neovascular retinopathies, though their best benefit is obtained when they are used in the early phases of the disease when the pathological condition has not yet fully manifested. In addition to disease prevention, diet supplements could also attenuate further complications due to the chronic use of specific drugs. Moreover, diet supplementation in combination with drug treatments might synergize with the pharmacological therapies by improving their effects in terms of efficacy and duration. However, nutraceuticals can also interact with other supplements or drugs, altering their pharmacological effects. Nonetheless, nutraceuticals providing an extra intake of natural molecules, such as polyunsaturated fatty acids (PUFAs), probiotics, and antioxidants, may also be useful in angiogenesis-associated ocular diseases [87]. Recently, the European Medicines Agency (EMA) has approved the use of the omega-3 eicosapentaenoic acid (EPA) as a drug (with the name ‘icosapent ethyl’, the high purity form of EPA methyl ester), elevating it from the previous state of a nutraceutical to that of a medicinal product (https://www.ema.europa.eu/en/documents/overview/vazkepa-epar-medicine-overview_it.pdf; last updated on March 2021). 

**Figure 3:**
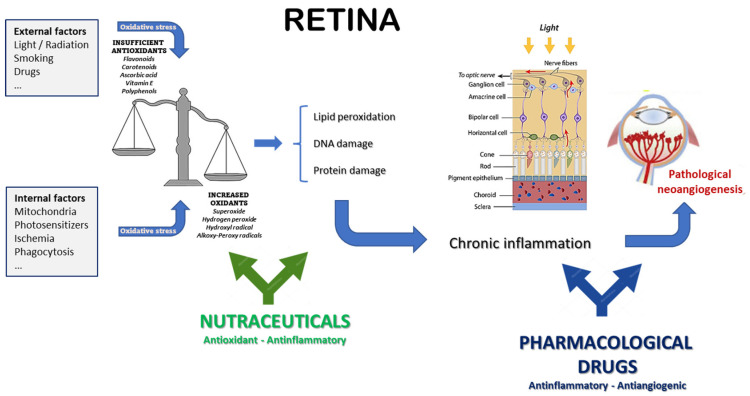
A schematic drawing illustrating the complementary and partially overlapping effects of nutraceuticals and pharmacological drugs on the pathways leading to neovascular retinal diseases.

Despite obvious limitations, preclinical and clinical studies strongly support the role of nutraceuticals in the management of sight-threatening retinopathies [25,88,89], as thoroughly reviewed in other recent publications [87,90,91]. Differently, in the present review, we aim to provide a pragmatic overview of the intersection between the use of pharmaceutical drugs and food supplements intended as nutraceuticals. In fact, pharmaceutical drugs mainly target the growth factors and cytokines responsible of neovessel growth, which are therefore downstream of the causative events. On the other hand, the vast majority of nutraceuticals advised for retinal pathologies take advantage of their anti-oxidative and anti-inflammatory properties (Figure 3), thus targeting with different efficiency the common upstream roots of most retinal diseases, especially in that part of the population at risk because of environmental or genetic factors [92]. Of course, there is no absolute or neat distinction between the different molecules, some of which might be endowed with more than one property, such as VEGF, which exerts both pro-inflammatory and pro-angiogenic effects [93]. Nonetheless, such a schematic view is helpful in understanding and describing the different and complementary activities of drugs and nutraceuticals. In this respect, integrating the natural endogenous antioxidant system with exogenous antioxidants should result in the attenuation of the pathological consequences of oxidative stress and inflammation, which are deeply involved in the pathogenesis of neovascular eye diseases. In fact, oxidative stress, inflammation, and angiogenesis are deeply interconnected [94], and may also act independently of the VEGF pathway [95]. Moreover, some nutraceuticals may also exert direct anti-angiogenic effects, possibly potentiating the effects of the classical anti-angiogenic drugs (mostly anti-VEGF) used to treat this kind of eye disease. 

Based on this knowledge, in this review, we aim to focus only on some possible ingredients, most of which are endowed with antioxidant, anti-inflammatory, and antiangiogenic properties. Blended together in different formulations, they might be useful in the prevention or treatment of the different neovascular diseases of the retina.

### 5.1. Association #1

For instance, a first possible association of nutraceuticals to strengthen the retinal defenses against AMD and possibly other neovascular retinal diseases could basically rely on different combinations of omega-3, cyanidins, and xanthophylls, specifically enriched with other specific natural components depending on the etiology and progression of the pathology under consideration. 

The Mediterranean diet has been proposed as a model of a healthy diet for the prevention of many diseases, including cardiovascular disorders, diabetes, and obesity [96,97]. It is rich in fish and vegetables, and with the enhancement of extra virgin olive oil, it has been associated with an over 40% reduced risk of diabetic retinopathy [98]. Several antioxidants and anti-inflammatory factors are present in the Mediterranean diet, such as phenolic antioxidants; gamma- and delta-tocopherols; tocotrienols; long-chain PUFAs, omega-6 and mostly omega-3, from which DHA and EPA are derived; different carotenoids, with lycopene being the most active; isothiocyanates from cruciferous vegetables; sulfur compounds from allium vegetables; and terpenoids. 

The function of many of these nutrients is carried out, at least partly, by the induction of the nuclear factor Nrf2 (nuclear erythroid factor 2-related factor 2), a transcription factor regulating the expression of more than 500 genes in the human genome, most of which have antioxidant and cytoprotective roles [99].

Animal studies have suggested that the oral intake of long-chain omega-3 PUFAs found in fish results in a two-fold increase in retinal long-chain omega-3 PUFAs, which may reduce the risk of diabetic retinopathy [100,101]. An adequate supply of long-chain PUFAs might prevent the low-grade chronic inflammatory process occurring in diabetic retinas, leading to microvascular alterations, glial proliferation, and the progressive apoptosis of retinal neurons [102]. A prospective study of 6 years enrolling 3482 Spanish participants aged 55 and older with type 2 diabetes found that the intake of 500 mg/day of dietary long-chain omega-3 PUFAs (found in at least two servings of fatty fish a week) was associated with a reduced incidence of severe DR in older adults [103,104]. These results were confirmed by another randomized, single-blind, controlled study addressing the utility of a combined therapy, in which monthly intravitreal injections of the anti-VEGF drug ranibizumab were coupled to daily treatments with oral omega-3 (DHA, 1050 mg/day). Results obtained on 33 subjects (42 eyes) with diabetic macular edema showed that this combined therapy resulted in a reduction of the central subfield macular thickness after 2 and 3 years of follow-up, as compared with ranibizumab alone. Such anatomical improvement was also paralleled by a trend of vision improvement [105,106]. 

The phospholipids present within photoreceptor membranes are highly enriched in omega-3 PUFAs, especially DHA. Therefore, a deficiency of omega-3 fatty acids (DHA and EPA) in photoreceptor membrane lipids, due to insufficient dietary intake, might contribute to drusen formation in the RPE and sub-RPE layers and to AMD pathogenesis [107]. We have used a mouse model system of AMD induced by subretinal injection of polyethylene glycol (PEG-400) to explore the effects of oral treatment with a complex mixture of fatty acids (commercially available as Macular-FAG^®^, Sooft Italia, Montegiorgio (FM), Italy) containing a balanced ratio of omega-3 and omega-6. Results have confirmed the beneficial effects of an adequate supplement of fatty acids, resulting in decreased complement-mediated inflammation, macrophage recruitment, and production of pro-inflammatory and angiogenic cytokines, finally leading to a functional rescue of retinal electrophysiology [108].

PUFAs in the retina are involved in the regulation of vascular function and angiogenesis, as indicated by a lipidomic analysis showing that the enzymatic activity of cyclooxygenases and lipoxygenases on omega-3 long-chain PUFAs generates metabolites that may inhibit inflammation and angiogenesis [109]. For instance, in a mouse model of OIR, the dietary intake of omega-3- and omega-6 PUFAs decreased the hypoxia-induced pathological neovascularization in the retina, reducing the avascular area in which vessel regrowth after injury was promoted. Interestingly, this model showed the opposite effects of omega-6 and omega-3 PUFAs. In fact, the intake of the former increased the microglial production of pro-inflammatory factors, while such an increase was prevented by the intake of omega-3 PUFAs, precursors of the mediators neuroprotectin-D1, resolvin-D1, and resolvin-E1, known to be potent inhibitors of neovascularization [100]. Indeed, the increased production of resolvins (a family of neuroprotector molecules derived from the omega-3 metabolites EPA and DHA) may attenuate the apoptotic events induced by mitochondrial and endoplasmic reticulum OS by reducing caspase-3 activity via AMPK activation [110]. Anti-angiogenic effects of similar importance as those obtained with omega-3 PUFAs were achieved in a mouse model of ischemic retinopathy with an inhibitor of VEGF [111], thus originally suggesting that the control of pathological retinal angiogenesis might benefit from a combined treatment with oral omega-3 PUFAs and injective anti-VEGF therapy.

The main clinical trial evaluating the role of nutraceuticals in AMD prevention has been the Age-Related Eye Disease Study (AREDS) [112]. In a follow-up study (AREDS-2), the effects of omega-3 were also evaluated, and it was reported that they did not appear to improve the efficacy of the AREDS diet [113]. This is in contrast with other preclinical and clinical studies showing beneficial effects of nutraceuticals enriched with omega-3 fatty acids [114,115]. In fact, a more recent post hoc analysis of those data found a correlation between omega-3 intake and the risk of nAMD development or progression [116]. Moreover, a very recent meta-analysis, including five observational clinical trials conducted in Europe, the USA, and Japan, including 12,068 AMD patients subdivided into four or five groups based on their fatty acid intake, found a protective association between dietary total omega-3 LC-PUFA intake, DHA intake, and EPA intake, and the incidence of nAMD [117]. 

Anthocyanins (anthos = flower and kyanos = blue) are a subgroup of flavonoids found in many typical foods of the Mediterranean diet, such as blueberries, eggplants, peaches, red oranges, figs, cherries, and olives. Dietary intake of anthocyanins may bring benefits to different brain functions, vision included. They are easily absorbed in the gastro-intestinal tract and may cross the blood–brain and blood–retinal barriers, as shown in pigs (mimicking human gastro-intestinal absorption) fed with blueberries, in which anthocyanins were detected in all examined tissues, including the brain and eyes [118]. The protective role of anthocyanins as antioxidants has been shown in a model system of rat retinal neurons damaged by an intraperitoneal injection of N-methyl-N-nitrosourea, in which anthocyanin-treated animals conserved a quasi-normal ERG response and showed little signs of gliosis [119]. In a different study on diabetic rats treated with blueberry anthocyanins, their antioxidant potency and protective effects were shown by the increased expression of HO-1 and the nuclear localization of the transcription factor Nrf2, finally resulting in increased expression of GSH and glutathione peroxidase, reduced levels of malondialdehyde (MDA) and ROS, and inhibition of lipid peroxidation through chelation of metal ions [120,121]. We have shown in a rat model of streptozotocin-induced DR that preventive supplementation with a mixture containing cyanidin-3-glucoside (C3G), verbascoside, and zinc was able to blunt both the oxidative stress due to high blood glucose and the further inflammatory events, finally rescuing the dysfunctional ERG [122].

Photo-oxidative stress is among the causes of AMD, and anthocyanins have been revealed to be an efficient protection against this type of damage [123]. Given their direct involvement in drusen formation, RPE cells are the preferred model in vitro to study the metabolic events that may lead to retinal degeneration and AMD. In such a model, for instance, it has been shown that photo-oxidative stress by UVB irradiation triggers apoptosis in ARPE-19 cells; however, anthocyanins (C3G) and xanthophylls (lutein and zeaxanthin) pre-treatment could attenuate this damage, thus suggesting that these polyphenols may be suitable as chemoprotective factors for the prevention of this kind of retinal damage [124]. Moreover, anthocyanins such as C3G contribute to the regeneration and de novo synthesis of the visual pigment rhodopsin, which is found in rod photoreceptor cells [125]. However, the inadvertent non-enzymatic metabolism of vitamin-A in the membranes of photoreceptor outer segments is known to generate photoreactive retinaldehyde-derived molecules (such as the diretinal fluorophore A2E), which are taken by RPE cells within phagocytosed outer segment disks and accumulate in RPE as lipofuscin. Upon irradiation with short-wavelength visible light, A2E acts as a photosensitizer, generating reactive oxygen species (ROS), which, in turn, lead to the degradation of A2E itself, and the generation of molecular fragments reacting with proteins, finally producing the advanced glycation end-products (AGEs) that are found in drusen. In vitro studies with RPE cells loaded with the diretinal fluorophore A2E and irradiated with short wavelength light have shown that C3G and quercetin were the only flavonoids among those tested able to decrease the cellular levels of ROS and at the same time also promote cell viability [126]. The antioxidant and anti-inflammatory effects of anthocyanins relevant to AMD prevention, have been further studied in vitro on RPE cells. In the case of oxidative damage induced by oxygen peroxide, the presence of anthocyanins was able to decrease the levels of reactive oxygen species and malondialdehyde and increase the levels of superoxide dismutase, catalase, and glutathione peroxidase, thus protecting RPE from apoptotic death. Moreover, anthocyanins decreased VEGF levels and activated Akt-signal pathways, with effects on the possible progression of AMD to neovascular disease [127]. The anti-inflammatory effects of C3G were addressed in a study in which RPE cells were treated with the potent inflammatory molecule 4-hydroxyhexenal (HHE). In this case, the cytotoxic effects triggered by HHE were significantly blunted by C3G pretreatment. In fact, C3G inhibited HHE-induced NLRP3 inflammasome activation and the further release of the proinflammatory cytokines IL-1β and IL-18 and the pro-apoptotic caspase-1, finally resulting in improved survival and proliferation of RPE cells [128]. Consistently, we have found that feeding rats with an association of C3G, verbascoside, lutein, and zinc resulted in stunning protection of the photoreceptor cells after the animals were exposed to very intense light (1000 lux cool-white light) for 24 h [129].

Proanthocyanidins derived from grape seeds have shown anti-angiogenic properties, being able to inhibit the migration, matrix metalloproteinase–2 and –9 secretion, and tube formation of human microvascular endothelial cells. These effects are mainly obtained through the inhibition of VEGF and angiopoietin-1 signaling [130]. 

Lutein and zeaxanthin are xanthophylls that easily cross the blood–brain and blood–retina barriers, so they are particularly dense in the macular region of the retina, giving it the characteristic yellow coloration, known as the macula lutea [131]. The fovea contains the highest concentration (approximately 13 ng/mm^2^), which is then decreasing towards the peripheral retina, which contains around 0.05 ng/mm^2^ [132,133]. The powerful antioxidant activity and the ability to filter blue light, responsible for photo-oxidative damage, are the main mechanisms by which these xanthophylls may prevent or attenuate the insurgence of retinal diseases. Feeding a diet without lutein to experimental animals gave rise to early degenerative signs in the retina, and patients affected by Macular Telangiectasia, a pathology leading to the absence of macular pigment, have a serious visual handicap, thus confirming the role and importance of the macular pigment [134]. In an in vivo study with diabetic rats, the treatment with zeaxanthin prevented the retinal damage typically associated with diabetes, decreasing nitro-tyrosine levels, DNA oxidative damage, and lipid peroxidation, finally bringing the retinal concentration levels of VEGF and the intercellular adhesion molecule-1 (ICAM-1: related to the inflammatory state of the tissue) to values similar to those of normal healthy controls [135]. Similar results were obtained with diabetic mice fed a lutein-enriched diet. Lutein did not affect the metabolic status of the diabetic mice, but restored normal ROS levels in the retina, preventing ERK activation and the decrease in BDNF, finally reducing RGC apoptosis and the consequent visual impairment [136].

In humans, it has been found that, like in the case of AMD, higher levels of lutein and zeaxanthin are associated with a significantly lower risk of DR [137]. Xanthophylls may also attenuate signs and symptoms of DR; a recent clinical study reported an improvement in glare sensitivity in patients with NPDR fed with a lutein supplement [138]. Another clinical study evaluated the benefits of lutein and zeaxanthin supplementation in type 2 diabetic patients already affected by NPDR and with a plasma concentration of these xanthophylls lower than normal at baseline. After 3 months, the plasmatic xanthophyll concentration was increased, in good correlation with a reduction of the macular edema and an improvement of visual acuity [139]. Along the same line, a randomized study on the effects of antioxidants on DR reported that lutein supplementation could delay DR progression within 5 years of observation [140]. The daily feeding of 10 mg lutein improved contrast sensitivity, glaring effects, and visual acuity in patients with nonproliferative DR [138]. 

Astaxanthin also belongs to the xanthophyll family, a ketocarotenoid derived by the oxidation of β-carotene, and commonly found as a pigment in algae, fish, and birds’ eyes. Astaxanthin is a powerful antioxidant, 80 times stronger than α-tocopherol and twice as strong as β-carotene [141,142]. Therefore, the possible use of astaxanthin in the treatment of chronic diseases involving OS, such as diabetes and its complications, has been addressed in some studies. The effect on ocular tissues of astaxanthin and lutein treatment in STZ-induced diabetic rats showed a significant decrease in OS and inflammatory cytokines resulting from NF-κB transcription activity, and increased amounts of antioxidant enzymes, with the final effect of preserving retinal architecture and function [143]. Other studies have shown that astaxanthin could modulate the glycemic state and decrease insulin resistance, also exerting anti-inflammatory and anti-angiogenic effects by decreasing the expression of NF-κB and TNF-α, finally inhibiting the expression of proinflammatory molecules such as ICAM-1 and VEGF [144]. In conclusion, the existing evidence indicates that the xanthophylls astaxanthin, zeaxanthin, and lutein may protect the retina from high-energy light radiation, and efficiently scavenge the oxidation byproducts, so their presence in nutraceuticals could be a good strategy to control the development of DR by reducing the oxidative damage to DNA, proteins, and lipids.

Finally, besides DR, the beneficial, primarily antioxidant effects of xanthophylls have been demonstrated for more eye pathologies, including AMD, age-related cataract (ARC), and uveitis [145]. Generally speaking, a constant supplementation of the xanthophylls lutein, zeaxanthin, and meso-zeaxanthin (forming the macular pigment), which the organism cannot synthesize and must be taken from food, is critical for the shielding and protection of the retina from continuous photo-oxidative stress [146]. As to their specific role in AMD, macular xanthophylls are also found within photoreceptor outer segment (POS) membranes, where they tend to concentrate in the membrane domains rich in polyunsaturated lipids [147]. Therefore, it is tempting to speculate that the specific accumulation of macular xanthophylls in the most vulnerable regions of photoreceptor membranes could play a relevant role in improving the antioxidant potential of these regions, and therefore preventing pathologies such as AMD [148,149]. Using a K.O. mouse strain for Sod2 (the gene for mitochondrial manganese superoxide dismutase) subjected to normal daily OS, it has been shown that zeaxanthin supplementation may blunt the dramatic consequences of such OS and preserve the structure of the RPE [150]. Clinical studies have also confirmed that food supplementation with lutein and zeaxanthin, as well as β-carotene, may delay the onset and progression of AMD, improving patients’ visual performance measured as contrast sensitivity, glare tolerance, and photo-stress recovery [6,151]. Most importantly, a clear correlation has been found between high blood levels of lutein and zeaxanthin and a significantly lower risk of AMD [152,153].

### 5.2. Association #2

A different association of nutraceuticals to increase retinal defense against oxidative stress damage and the side effects on the retina of diabetic patients could be based upon epigallocatechin-gallate (EGCG), resveratrol and curcumin.

Epigallocatechin-3-gallate (EGCG) is the predominant catechin-based flavonoid in green tea extracts [154]. EGCG has been proven to possess antioxidant, anti-inflammatory, neuroprotective, cardioprotective, and anti-carcinogenic effects [155,156]. Moreover, due to its low molecular weight and hydrophilic properties, EGCG can cross the blood–retinal barrier, thus homing into different ocular tissues, including the retina. This suggests that systemic EGCG administration might protect against retinal degenerative or neurodegenerative diseases [157]. In fact, EGCG has been shown to provide retinal protection against oxidative damage [158], ischemia [159], N-methyl-D-aspartate [160], and diabetic retinopathy [161]. When the human RPE cell line ARPE19 was exposed to UVA-induced damage, the presence of EGCG enhanced their survival by blunting the production of ROS and the activation of the pro-inflammatory mediators MAPK and COX-2 [162], and stimulating the expression of Nrf2, the transcription factor responsible for the endogenous production of antioxidants [163].

EGCG has long been associated with angiogenesis inhibition [164]. EGCG is a potent inhibitor of metalloproteases [165,166], which are used by endothelial cells to migrate through tissues to form new blood vessels. Moreover, EGCG may inhibit VEGF production by blocking the induction of VEGF transcription, inhibiting both the activation of Erk-1 and -2 and the binding of the transcription factor AP-1 to the VEGF promoter [167]. These same mechanisms, inhibition of the metalloprotease MMP9 and VEGF expression, have also been implied in angiogenesis inhibition by EGCG in eye models of neovascularization. VEGF-induced proliferation and tube-formation of human microvascular retinal cells in vitro were inhibited by EGCG, as were corneal neovascularization and vascular leakage induced by alkali burns [168]. Later, it was shown that EGCG inhibited cell migration and capillary tube formation by HUVEC cells in vitro through the inhibition of the PI3K/AKT and MEK/ERK pathways, which acted synergistically to induce activation of the FOXO transcription factors, thus triggering the antiangiogenic effects of EGCG [169]. These pathways have also been implied in EGCG protection of retinal vascular endothelial cells from high-glucose-induced retinopathy [170]. More recently, in a mouse model of laser-induced CNV simulating the neovascular stage of AMD, oral treatment with a pro-drug of EGCG significantly alleviated mouse laser-induced CNV leakage and reduced CNV area by down-regulating the HIF-1α/VEGF/VEGFR2 pathway [171]. 

Among the non-flavonoid polyphenols, resveratrol (3,5,4′-trihydroxy-trans-stilbene), a polyphenolic antioxidant belonging to the stilbene family and commonly found in grape skin and seeds, and curcumin (1,7-bis(4-hydroxy-3-methoxyphenyl)-1,6-heptadiene-3,5-dione) have shown protective effects in DR and AMD models. 

The broad spectrum of anti-inflammatory and neuroprotective properties of resveratrol has been shown in several scientific reports to exert a beneficial effect on eye tissues [172,173]. Studies in vitro with bovine retinal capillary endothelial cells stressed by high glucose have shown that resveratrol treatment may prevent ROS-induced apoptosis partly by its antioxidant activity, and partly through the activation of the AMPK/Sirt1/PGC-1α pathway [174]. The antioxidant effects of resveratrol are predominant in a model of chemically mimicked hypoxia induced by cobalt chloride treatment of the human pigmented epithelium ARPE-19 cell line, in which the reduction of oxidative stress also led to a decreased production of pro-angiogenic and pro-fibrotic factors [175]. However, when the oxidative stress on ARPE-19 came from high glucose treatment, the presence of resveratrol resulted in the sequential inhibition of the stimulated secretion of inflammatory cytokines (IL-6 and IL-8) and of the activation of TGFβ, PKCβ, and COX-2, also preventing the downregulation of connexin 43 (involved in gap junctions) [176]. Studies on STZ-induced diabetic rats brought to attention the induction of paraoxonase-1 (PON1) in the retina as an intermediary in the protective effects exerted by resveratrol, with anti-inflammatory effects reverberating on the reduction of retinal vascular permeability and inhibition of pro-apoptotic caspase-3 activity [177]. 

Ultraviolet radiation, although weakly absorbed by protein and DNA, may penetrate the lens and reach the retina, triggering the production of reactive oxygen species (ROS), and finally inducing OS in RPE cells, thus contributing to the development or progression of AMD. Resveratrol has been shown to protect ARPE-19 cells from UVA-induced cell damage by blunting ROS production and preventing the activation of inflammatory pathways [178]. In a mouse model of neovascular AMD obtained by laser photocoagulation to induce CNV, oral supplementation with resveratrol could prevent the development of choroidal neovascularization. Such an effect was due to the inhibition of macrophage infiltration into the RPE-choroid and the suppression of inflammatory and angiogenic factors [179]. Interestingly enough, from the perspective of combined treatment with drugs and nutraceuticals, a study in vitro with RPE cells has suggested that the concomitant presence of resveratrol could mitigate the adverse effects of anti-VEGF agents on RPE cells [180].

Besides its many different properties, resveratrol can also inhibit neovascular proliferation. It has been shown in a model of corneal neovascularization in mice induced by the topical implantation of sponges releasing VEGF and FGF2. When resveratrol was fed to mice in drinking water for three days before sponge implantation, the area and density of neovascularization induced by the angiogenic factors were sensibly reduced, indicating that active amounts of resveratrol could reach the cornea after the gut’s absorption [181]. Resveratrol has a direct effect on human vascular endothelial cells in vitro, inhibiting their growth and motility, their tube-formation ability, and their production of the metalloprotease MMP2, used for local invasion in the ECM to make space for the formation of new blood vessels [182]. Moreover, resveratrol directly inhibits the secretion of VEGF-A from human retinal pigmented epithelial cells [183]. In a mouse model of macular telangiectasia (MT) characterized by abnormal neovascular proliferation of the retinal vasculature surrounding the fovea, the neovascular lesions were partially due to the increased expression of VEGF. Upon oral administration of resveratrol, given either starting 5 days before the appearance of the neovessels or 6 days after this event, a consistent reduction of neovascularization was observed, likely due to a decreased expression of VEGF [184]. 

Curcumin is endowed with several useful beneficial properties, including anti-inflammatory, antioxidant, antimicrobial, and anticancer activities. The anti-inflammatory and antioxidant properties of oral curcumin have been specifically exploited in the treatment of several eye diseases [185]. In DR model systems, curcumin treatment resulted in an increase in antioxidant enzymes, upregulation of HO-1 and nrf2, reduction or inhibition of inflammatory mediators and growth factors, and inhibition of proliferation and migration of retinal endothelial cells, as evaluated in the different cell lines HRPC, HREC, and ARPE-19. In AMD experimental model systems, curcumin treatment resulted in the reduction of ROS, inhibition of apoptosis mediators and cellular inflammatory genes, and upregulation of the protective enzymes HO-1, thioredoxin, and NQO1 [186,187]. Clinical reports also corroborate the use and efficacy of curcumin in the treatment of ophthalmic pathologies [188]. However, with the main caveat of its poor bioavailability, which implies treatment with relatively high doses [189], the use of curcumin as a food additive has been approved by the WHO at a maximum daily dose of 3 mg/kg [190], although clinical trials have shown the safety of curcumin up to 12 g/day [191]. The protective antioxidant and anti-inflammatory activities of curcumin (10 µM) have been verified on different eye-derived cell lines exposed to simulated hyperglycemic conditions. The effects were a significant blunting of ROS concentration in RPE cells and a decrease in TNF-α release in human retinal endothelial cells (HREC); retinal pericytes were also protected from high-glucose damage [192,193]. Curcumin treatment (10 µM) of HREC exposed to high glucose triggered an increase in HO-1 expression, a stress response protein that is induced by oxidative or heat stress [194]. This mechanism may suggest a double role of curcumin as an antioxidant, both direct and indirect, through the activation of the transcription factor Nrf2, which can now move into the nucleus, thus promoting the transcription of genes encoding antioxidant enzymes such as HO-1 [195,196]. STZ-induced diabetic rats were treated with curcumin by different routes. The intraperitoneal daily injection of 80 mg/kg could prevent, on the one hand, the retinal increase in the OS marker malondialdehyde (MDA) and, on the other hand, the decrease in antioxidant GSH levels [197]. The administration of oral curcumin at 1 gr/hg resulted in a decrease in blood glucose and the prevention in the retina of the increase in the proinflammatory cytokines TNF-α and VEGF. These events were accompanied by an increased antioxidant level of superoxide dismutase (SOD), catalase, and GSH, and a lesser increase in the oxidative protein damage marker nitro-tyrosine, and the oxidative DNA damage marker 8-hydroxy-20-deoxyguanosine [198,199]. 

Curcumin is also a pleiotropic angiogenesis inhibitor; it inhibits the expression of two major angiogenesis factors, VEGF and bFGF, and may decrease nitric oxide generation in endothelial cells [200,201]. Moreover, curcumin binds to CD13/aminopeptidase-N (APN), a membrane-bound enzyme found in blood vessels undergoing active angiogenesis, and blocks its activity, thereby inhibiting angiogenesis. [202,203]. The efficacy of curcumin in proliferative retinal diseases has been thoroughly studied and reported [187].

In neovascular AMD, the overactivation of the Wnt/β-catenin signaling pathway triggers several downstream mechanisms, finally resulting in increased inflammation, OS and CNV. Curcumin has been shown to inhibit the Wnt/β-catenin signaling, thereby affecting OS, inflammation, and angiogenesis in exudative AMD [204]. Supportive clinical data were obtained from a retrospective study enrolling 18 naive patients who received intravitreal injections of anti-VEGF and daily oral treatment with a curcumin-based nutritional supplement vehiculated by enterosoma-I^®^, and 24 naive age-matched controls with the same diagnosis undergoing only intravitreal injections. Data analysis has shown a clear improvement (*p* < 0.05) of the median best-corrected visual acuity in treated patients vs. controls, but no statistical difference in central macular thickness between groups (*p* > 0.05). However, the total number of intravitreal injections was significantly (*p* < 0.05) reduced in the curcumin group as compared to controls (4 vs. 7 median values). The treatment with the curcumin-based nutritional supplement resulted in safety; hence, curcumin could be an effective adjuvant of anti-VEGF treatment, improving functional outcomes and prolonging the duration of intravitreal therapy [205].

### 5.3. Association #3

Feeding nutraceuticals to newborns is a very delicate matter. However, in the case of premature birth, when the newborn is at high risk of developing an oxygen-dependent retinopathy like ROP, reinforcing the natural defenses of the retina with PUFAs, xanthophylls, and carotenes could be very advisable.

Lutein and zeaxanthin are normally transferred from the mother to the baby. Most of this takes place during the last trimester of pregnancy and during lactation after birth. Therefore, preterm infants do not have enough of these pigments, which may protect their developing retina from the intense OS contributed by the oxygen shifting conditions to which they are subjected. However, in spite of the well-documented protective effect of lutein, it remains controversial whether its supplementation may prevent or attenuate the occurrence of ROP in preterm infants. A recent meta-analysis including data from three RCTs with a total of 406 participants failed to show any reduction in ROP incidence [206]. On the contrary, when tested in an experimental OIR mouse model, lutein was able to reduce vascular edema, restore the retinal vascular bed, and promote neuroprotection [207]. Such a difference between the human and mouse experimental data might be due to the different absorption characteristics of the two species. Lutein is a lipophilic molecule, so its absorption and bioavailability are variable. By changing the vehicle in which it is dissolved and using vegetable-derived or fish oil to dissolve lutein, its bioavailability in mice could be enhanced [208,209]. 

Premature infants have impaired synthesis of LC-PUFAs from precursors, and because of the loss of placental and maternal growth factors at birth, they may require preformed DHA and Arachidonic Acid (AA) [210], which are critical nutrients for the maturation of a functional retina. Indeed, a randomized, double-blind, controlled trial on 160 premature infants with gestational age lower than 32 weeks and a birth weight < 1500 g at risk of ROP found that a daily supplement of 300 mg omega-3 was associated with a significant reduced risk for ROP [211]. The importance of fatty acid supplementation in preterm infants was further confirmed by a study on 175 very-low-birthweight babies, concluding that higher mean daily serum levels of DHA during the first 28 postnatal days correlated with less severe ROP even after adjustment for other known risk factors, but only in infants with sufficiently high arachidonic acid (AA) levels [212]. In fact, a further study by the same group on 206 infants born at less than 28 weeks’ gestation and supplemented with an enteral oil providing AA (100 mg/kg/d) and DHA (50 mg/kg/d) (AA:DHA group) or no supplementation within 3 days after birth found that enteral AA:DHA supplementation resulted in overall higher serum levels of both AA and DHA and a 50% lower risk of developing severe ROP [213]. Finally, with the aim of exploring the efficacy of different sources of omega-3 fatty acids, a retrospective clinical study on 96 preterm infants compared the use of fish oil-containing lipid solutions with soybean-based lipid solutions. Results suggested that the use of the former was associated with a lower incidence of ROP and a decreased need for bevacizumab treatment in preterm infants [214].

Vitamin A is a critical factor in the development of the retina in newborns, and its deficiency may cause serious problems during its maturation, which happens after birth. A clinical study on 262 extremely preterm infants has shown that vitamin A supplementation is associated with a decreased incidence of Type 1 ROP [215]. These results were confirmed by another study on 62 preterm very-low-birthweight newborns, showing that vitamin A oral treatment resulted in a decrease in incidence and severity of ROP cases, and no babies receiving vitamin A had to be treated by laser photocoagulation [216].

### 5.4. Further Elements in the Associations

The presence of some vitamins and microelements might add further strength to each of the previous associations.

Vitamins and microelements are necessary for the correct metabolic functioning of the organism, and some of them are also endowed with antioxidant potential. They also have a relevant role in the pathogenesis and treatment of ophthalmic neovascular diseases.

Vitamin B1 (thiamine) is a strong scavenger of free radicals and is involved in the regulation of intracellular glucose, thus preventing the activation of the polyol pathway typically induced in diabetic patients by the hyperglycemic stress [217,218]. Moreover, high serum levels of thiamine may protect the microvascular endothelium from damage due to AGEs. Therefore, high doses (50–100 mg/day) of thiamine supplementation may exert neuroprotection, and reduce the risk of diabetic retinopathy and nephropathy [219]. Vitamin B2 (riboflavin) supplementation in humans improves the synthesis of L-methylfolate, thus reducing homocysteine (Hcy) levels and lowering blood pressure [219]. Hcy levels can be decreased also by vitamin B6 and B12 supplementation, whereas high doses of vitamin B3 (niacin) may enhance diabetes and increase the risk of macular edema [219].

Vitamin C is a water-soluble vitamin existing in two main forms, ascorbic and dehydroascorbic acid, and is necessary for the regeneration of other antioxidants, such as vitamin E and glutathione [220,221]. It can treat primary hypertension [222] and capillary endothelium dysfunction in diabetic individuals [223]. A 10-fold lower level of ascorbate in the vitreous humor (compared to healthy controls) has been reported in diabetic patients with a higher incidence of proliferative DR and diabetic macular edema [224]. The association of vitamin C and statins may decrease the risk of nonproliferative DR in a dose-dependent manner, better than statins alone [225]. Finally, vitamin C may also have a role in angiogenesis. A study conducted on human umbilical vein endothelial cells (HUVECs) has shown that vitamin C may counteract the effect of VEGF on the increased permeability of the blood–retinal barrier, thus contributing to reducing the risk of macular edema in DR [226].

Vitamin D is necessary for insulin release and tissues responses to it, control of the inflammatory response, and vascular health [219]. The right amount of vitamin D is essential to decreasing the risk and severity of DR and its progression towards the neovascular form [227].

Vitamin E (a potent antioxidant) supplementation (1800 IU daily) in patients with less than 10 years of type 1 diabetes resulted in a significant improvement in retinal blood flow [228]. The elevated oxidative stress that is present in diabetic patients is reduced by vitamin E treatment [229]. The beneficial effects of vitamin E supplementation are increased by its association with vitamin C [230]. The role and efficacy of vitamin E in the prevention and treatment of clinical ROP have been known for several decades [231,232]. In a recent study, the topical administration of eye drops containing an association of Vitamin E and Coenzyme Q10 to 110 newborn babies with type 1 ROP resulted in a milder development of ROP, so that a significantly lesser number of babies had to undergo laser photocoagulation treatments to stop abnormal vessel proliferation, as compared to 131 babies not receiving the topical treatment [233]. The role of vitamin E in AMD biogenesis could be evidenced by the fact that its deprivation in the diet leads to the accumulation of extracellular debris in the form of lipofuscin [234] and to retinal damage [235], although the loss of photoreceptors after rats’ chronic exposure to bright light could not be significantly attenuated by a supplement of vitamin E [236]. However, high dietary intakes of vitamin E have been clinically correlated with a slower progression of AMD [237].

Results from the AREDS studies I and II suggested that vitamins A, C, and E could decrease the risk of macular degeneration development [112,113]. Consistently, high dietary consumption of foods rich in provitamin A and vitamin C has been correlated with favorable AMD outcomes [238,239]. Moreover, results from the AREDS Study Group have shown that the likelihood of neovascular AMD proportionally decreases with higher intakes of vitamin C [240]. However, despite several clinical studies addressing the potential benefits of vitamins A, C, and E, their efficacy on the progression of AMD is still uncertain and a matter of discussion [6]. Differently, the role of vitamin D in AMD prevention seems to be sufficiently clear, though the effective amount to be used in nutraceuticals is still under investigation [241]. 

Carotenoids are naturally occurring pigments that give fruits and vegetables their yellow/orange color. Humans must acquire carotenoids from their diet because they are not synthesized by human cells. The presence of carotenoids in the retina (as well as in other tissues) is critical for its metabolism and defense against OS. In fact, these natural antioxidants contribute to the quenching of free radicals produced by physical or metabolic reactions, thus protecting the eye from oxidative stress, apoptosis, mitochondrial dysfunction, and inflammation. Beta-carotene in the body is converted into vitamin A (retinol), which is important for good vision and eye health, a strong immune system, and healthy skin and mucous membranes [242]. However, getting high amounts of either vitamin A or beta-carotene from nutraceuticals (but not from foods) can be dangerous for people who smoke [243].

Trace elements play specific roles in the pathogenesis and progression of diabetes and its complications, and their deficiency could be relevant in diabetic patients [244].

Zinc is a dietary microelement and a necessary cofactor for DNA synthesis and cell replication, immune functions, and the metabolism of carbohydrates and proteins. The progression of chronic pathological conditions, including diabetes and its microvascular complications such as DR, has been associated with zinc deficiency [245] and a concomitant increase in copper [246]. The dietary role of zinc has been recognized by the EFSA, and it can be officially claimed in nutraceuticals [247]. 

High doses of oral zinc supplementation may slightly decrease morbidity and mortality in preterm neonates and improve short-term weight gain and linear growth. However, not enough data are available to establish an effect of zinc supplementation on long-term neurodevelopment and ROP [248,249]. 

Trivalent chromium (Cr3+) is required for normal glucose metabolism. Although there is no clear evidence that diabetic patients suffer from chromium deficiency, it is known that this condition, experimentally generated, leads to reduced glucose tolerance, which can be improved by the addition of chromium to the diet [250]. Chromium appears to act by enhancing insulin action by increasing the number of insulin receptors and their binding to insulin [250,251]. The most important evidence of the role of chromium supplementation in type 2 diabetes comes from a randomized, double-blind, placebo-controlled study conducted on a Chinese population [252].

Zinc and copper added to nutraceuticals contribute to the decrease in AMD progression risk, suggesting that these microelements play important roles in the homeostasis of retinal health [253]. More specifically, zinc antioxidant properties may efficiently blunt the risk of AMD and visual impairment, likely by modulating the synaptic transmission elicited by light excitation of rhodopsin and controlling the undesired complement activation triggered by drusen accumulation [253]. Selenium is another efficient antioxidant agent, also contributing to the activity of GSH antioxidant defense, and may thus inhibit the oxidative damage of membrane lipids and, in turn, reduce the risk of AMD [254]. Moreover, the exclusion of vitamin E and selenium from the diet of laboratory animals determined a relevant decrease in total PUFAs in RPE and in retinal rod outer segments, which could be recovered by adding back the missing microelements to the diet [255].

### 5.5. Role of the Gut’s Microbiota

The influence of dietary habits and nutraceuticals on eye disease could also work in some indirect ways, influencing the nature and state of the gut microbiota [256]. Experimental and clinical observations have demonstrated the influence of a gut-retina axis on ocular pathologies, showing that aberrations in the gut microbiota can be associated with ocular diseases in both humans and animals. Cellular and antibiotic therapies, intermittent fasting, and an altered diet have been shown to restructure the gut microbiota and alter the occurrence of some ocular pathologies [257]. Alterations in the gut microbiome may influence the host immune response, thereby suggesting that immunopathogenesis could be the basis for the link between intestinal dysbiosis and ocular diseases [258]. Most recently, Morita et al. demonstrated in preclinical studies that Lactobacillus paracasei KW3110 (a lactic acid bacterium) may activate M2 macrophages (macrophages associated with anti-inflammatory reactions), thus suppressing blue light-induced retinal inflammation both in vitro and in vivo, and mitigate age-related chronic inflammation by modulating gut microbiota composition and immune system functions in aged mice, thereby reducing age-related retinal ganglion cell (RGC) loss. These results suggest that L. paracasei KW3110 may have a preventive effect against degenerative retinal diseases, including AMD [259,260]. The role of gut microbiota in the development and progression of AMD has been further described in a recent comprehensive review [261]. Similarly, the determinant role of the gut-retina axis and the effects of the microbiota in diabetic retinopathy are emerging in several studies of recent publication [262,263,264].

### 5.6. Final Considerations

Finally, we must keep in mind that the effects of nutraceuticals are not restricted to the eye only but influence the metabolism of the whole body. In fact, Ludwig Feuerbach is quoted as having said, “We are what we eat” [265]. Indeed, as heterotrophic organisms, we must take from food all the necessary nutrients and energies to build our body and keep it healthy in a homeostatic equilibrium [266]. Pathologies occur with the breaking of this homeostatic equilibrium, either because external noxious agents determine such events, and/or because our habits and behaviors make our organism more susceptible to the deleterious effects of aging, which means a decreased defense against oxidative stress. Eye pathologies are, of course, included. As Prof. Josef Flammer said a few years ago when talking about glaucoma, eye pathologies are usually not restricted to the ocular district alone but may derive from the involvement of the whole body [267]. Our diet nowadays is mainly based on lifestyle habits rather than physiological needs, so the intake of some nutrients is often insufficient to maintain the right metabolic equilibrium, preserving the function of all tissues and organs over time [268,269]. Moreover, not all the bioactive molecules present in edible plants are represented in foods usually eaten by different populations. Therefore, the more we learn about the links between metabolism and pathologies, and the role that nutrients have in controlling this interaction, the more we realize that a correct integration of our diet with defined nutrients could help in restoring some physiological functions altered by a wrong balance between anabolism and catabolism. Indeed, the use of nutraceuticals is becoming increasingly accepted by physicians and their patients for the prevention of certain pathologies, or even their early phase treatments. Our goal in this review has been to give further support to the relevant role of nutrients specifically in the field of ocular pathologies, mainly those that pose the most relevant sight-threatening risk due to the neovascular invasion of the macula and for which no cure is available yet. We believe that the available pharmacological treatments based on anti-VEGF strategies could also benefit from the addition of rightly chosen nutraceuticals, based on the data presented here. Accordingly, clinical evidence exists to support this point of view. In a clinical study, patients with active neovascular AMD under treatment with anti-VEGF were randomly divided to receive a nutraceutical containing a mix of antioxidants, zinc, and carotenoids with or without omega-3 fatty acids (docosahexaenoic acid and eicosapentaenoic acid), and compared to naïve patients treated with only anti-VEGF. Results showed that omega-3 supplementation combined with anti-VEGF treatment is associated with decreased vitreal VEGF-A levels in wet AMD patients [270]. Along the same line, another clinical study enrolled 60 patients with treatment-naïve CNV, who were randomized into three groups: aflibercept monotherapy (AM), aflibercept plus pranoprofen (AP), or aflibercept plus nutraceutical (AN) tablets containing multivitamin antioxidant and mineral supplementation plus omega-3. After 12 months of treatment, results showed that compared with AM, each combination group showed an improvement, although no significant benefits in BCVA were found over AM. More specifically, nutraceutical support with omega-3 led to a decreased need for intravitreal injections [271]. Moreover, therapeutic intravitreal drug injections can be given only a few times per year, while nutraceuticals can be used on a daily basis, so that their presence in the organism and their function are continuous and may improve patients’ responses to therapy while also alleviating its side effects. For instance, resveratrol in vitro has been shown to be able to prevent and even reverse the negative effects on pigmented epithelial cells exerted by treatment with the anti-VEGF bevacizumab [180]. Of course, more ad hoc clinical trials are necessary to prove this hypothesis, so pharmaceutical companies, hospitals, and eye clinics are encouraged to try new protocols based on this philosophy in order to verify whether there could be a real and useful contribution by nutraceuticals to the pharmacological treatment of neovascular retinal pathologies.

From a pragmatic point of view, considering their rationale for use in prevention and early treatment, the clinical application of nutraceuticals is still limited by elements, the most important of which are the necessity of a clear evaluation of individual risk factors and better methods for early detection of the pathology. Additional limitations include (1) the effective bioavailability of nutraceuticals, including their PK and PD in the eye; and (2) the interaction between drugs and nutraceuticals. In fact, drugs can influence the patient’s nutritional status, body weight and absorption of nutrients, while nutraceuticals and herbal extracts may significantly alter the drug’s effects and efficacy either directly (for instance, molecules contained in a nutraceutical may modify the effect of the drug), or indirectly, by altering the absorption or metabolism of the drug; (3) the lack of a strong clinical evidence for nutraceutical efficacy in patients, which has been shown mostly in preclinical studies using acute pathology models raised in rodents, which do not exactly replicate the human pathology, and have a different pharmacokinetics and pharmacodynamics of the compounds under exploration. Therefore, appropriate clinical studies with long observation times are strongly needed in order to demonstrate the effects of nutraceuticals on the generally slow-progressing eye diseases, always keeping in mind the difficulties of such clinical trials in their translation into clinical practice (Table 1).

## 6. Conclusions

Here we have presented a panoramic view of the possible treatments available nowadays for sight-threatening proliferative neovascular retinal diseases. 

**Figure 4:**
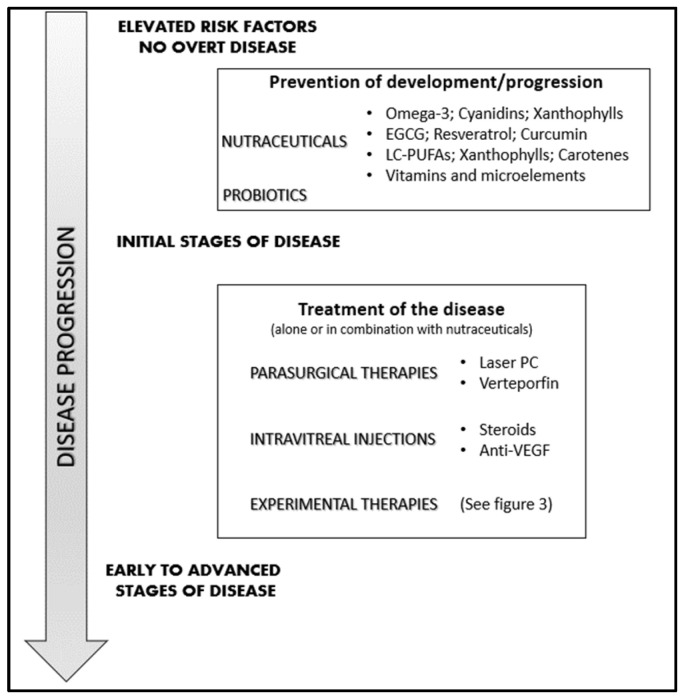
A schematic representation of the preventive and therapeutic approaches presented in this review.

The emerging general picture tells us about two possible approaches that are not mutually exclusive (Figure 4). 

The pharmaceutical approach, which can be coordinated with a para-surgical treatment, is mainly based on the use of intravitreal drugs that block the angiogenic stimulation by VEGF, as described in the first part of this review. It has been shown to be a treatment with a good percentage of success, although not devoid of side effects. Such therapy is given when the pathology is already manifest, with the aim of slightly improving or conserving the visual acuity and halting or delaying the progression of the pathology. Much better would be an approach that could estimate in a still-healthy patient the risk of developing a neovascular retinal disease, thus intervening with mild, non-invasive treatments and improving or integrating the endogenous defenses in the ocular district. Such an approach is possible based on the anamnesis of the patient and a risk factor analysis derived from the epidemiological knowledge of these diseases. On this basis, a choice of nutraceuticals with proven efficacy in retinal defense could be advised to the patient, with the aim of preventing or at least delaying the insurgence of the pathology. In this respect, pharmaceutical drugs tend to be curative, and usually target pro-angiogenic and inflammatory factors, thus inhibiting neo-vessel proliferation. Nutraceuticals are mostly antioxidant and anti-inflammatory, so they act upstream of the induction of damage leading to the retinal disease, thus working better in the prevention and limitation of the damage. The presence of anti-inflammatory effects in both classes might synergize if they are given together.

In conclusion, given the absence of other treatments working in the prevention or early therapy of neovascular eye pathology, the sight-threatening relevance of such pathologies, and the relative innocuity of nutraceutical treatments, we believe that, on a patient-by-patient basis, the administration of nutraceuticals, either alone or in association, could benefit many patients, delaying the progression of their disease and likely improving the efficacy of pharmaceutical drugs.

## Figures and Tables

**Table 1:** Critical points to be addressed in future research.

Elaborate contingency tables to establish the relative individual risk of developing NV retinal diseases based on genetic and environmental criteria
2. Improve the analytical methods for early diagnosis
3. Improve the formulation and the bioavailability of nutraceuticals
4. Improve the understanding of the possible interactions between nutraceuticals and drugs used in the treatment of NV retinal diseases
5. Run more RCT to show the efficacy of nutraceuticals and their possible role as coadjuvant in pharmacological therapies of NV retinal diseases

## Data Availability

No new data were created in this bibliographic research.
